# Supplementary material for: Loss of 5-methylcytosine alters the biogenesis of vault-derived small RNAs to coordinate epidermal differentiation
Source: Nat Commun. 2019 Jun 11;10:2550. doi: 10.1038/s41467-019-10020-7 (PMC6560067; doi:10.1038/s41467-019-10020-7)
Supplement: Supplementary file 1 — Supplementary Information [file 41467_2019_10020_MOESM1_ESM.pdf]

*Supplementary Information*

# Loss of 5-methylcytosine alters the biogenesis of Vault-derived small RNAs to coordinate epidermal differentiation

Sajini et al.

## Supplementary Figure 1

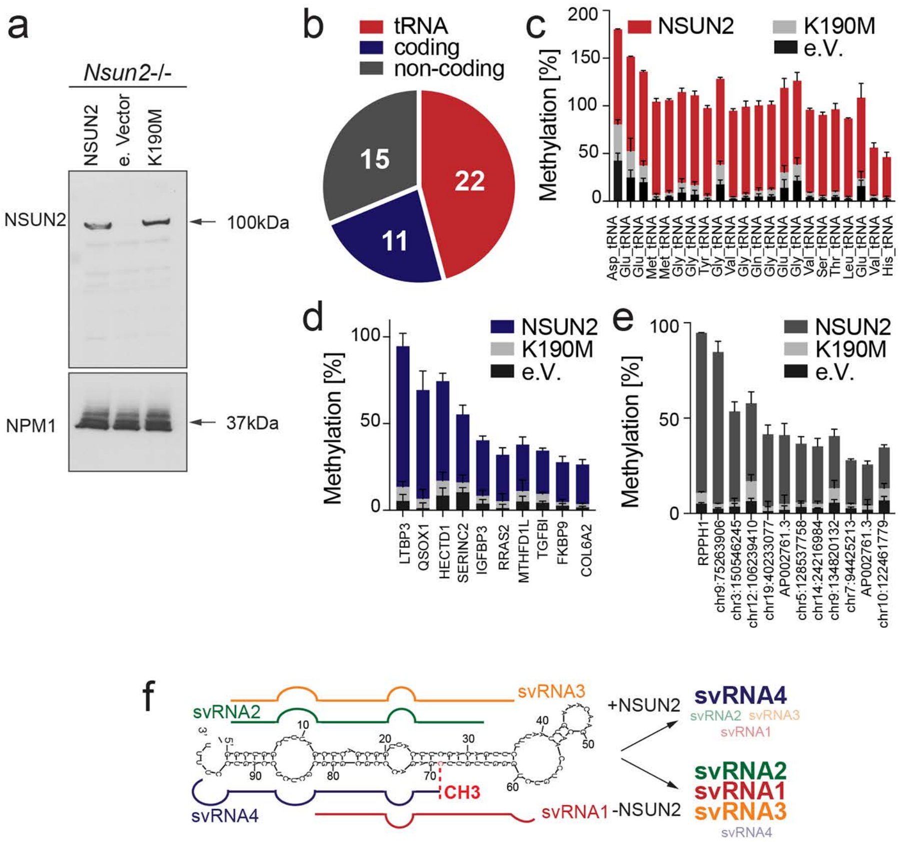


**Supplementary Figure 1. NSUN2-dependent RNA methylation.** (**a**) Western blot detecting NSUN2 in *NSUN2*-/- cells infected with the empty (e.) vector (ctr), the enzymatic dead construct K190M, and the wild-type NSUN2 construct. (**b**) Number of NSUN2-dependent methylation sites in tRNA (red), coding (blue) and non-coding (grey). (**c-e**) Methylation of tRNA (c), coding RNA (d) and non-coding RNA (e) in *NSUN2*-/- cells infected with the empty (e.) vector (ctr), the enzymatic dead construct K190M, or the wild-type NSUN2 construct. (**f**) Schematic illustration of NSUN2-dependent methylation (CH3) of VTRNA1.1 and the small regulatory non-coding fragments svRNA1-4. Error bars indicate s.d. (n = 5 bisulfite conversion assays) (c-e). Source data are provided as a Source Data file.

# Supplementary Figure 2

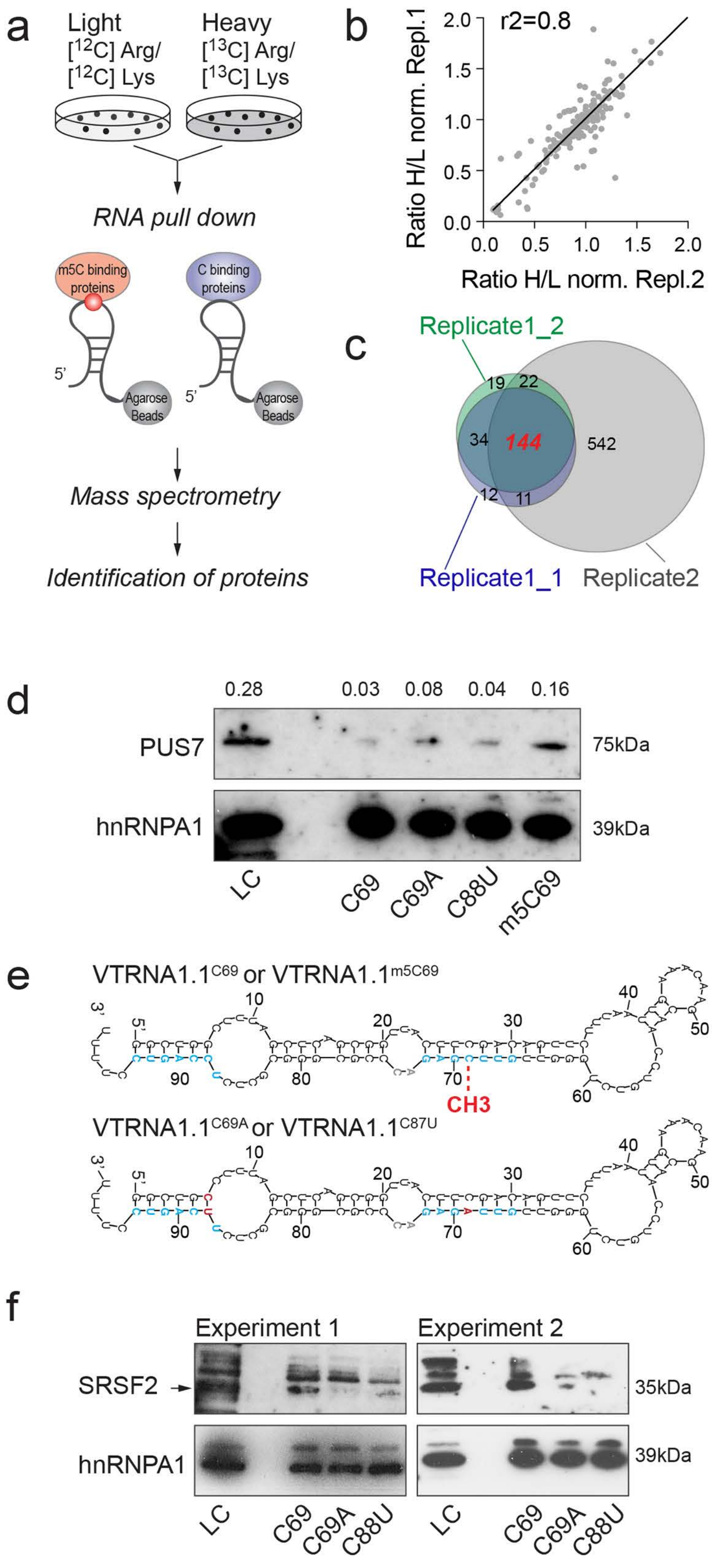

**Supplementary Figure 2. SRSF2 and PUS7 bind methylated VTRNA1.1. differentially. (a)** Schematic illustration of the SILAC-based quantitative mass-spectrometry assay. (**b**) Correlation of differential binding of proteins to VTRNA1.1 in two technical replicates. (**c**) Venn diagram showing a total 144 commonly identified proteins in two independent replicates. (**d**) RNA pull-down of methylated ($m^5$C69) and unmethylated (C69) VTRNA1.1 or VTRNA1.1 carrying the indicated point mutations followed by Western blot for PUS7. Numbers indicate band intensity (ImageJ). (**e**) Human VTRNA1.1-constructs with highlighted (blue) putative SRSF2-binding sites (upper panel) and the constructs carrying the point mutations to interrupt the binding sites (lower panel). (**f**) RNA pull-downs using wildtype or mutated (C69A; C88U) VT-RNA1.1-constructs confirm both putative SRSF2 binding sites are necessary for SRSF2 binding. hnRNPA1 served as a loading control in (d, f). Source data are provided as a Source Data file.

# Supplementary Figure 3

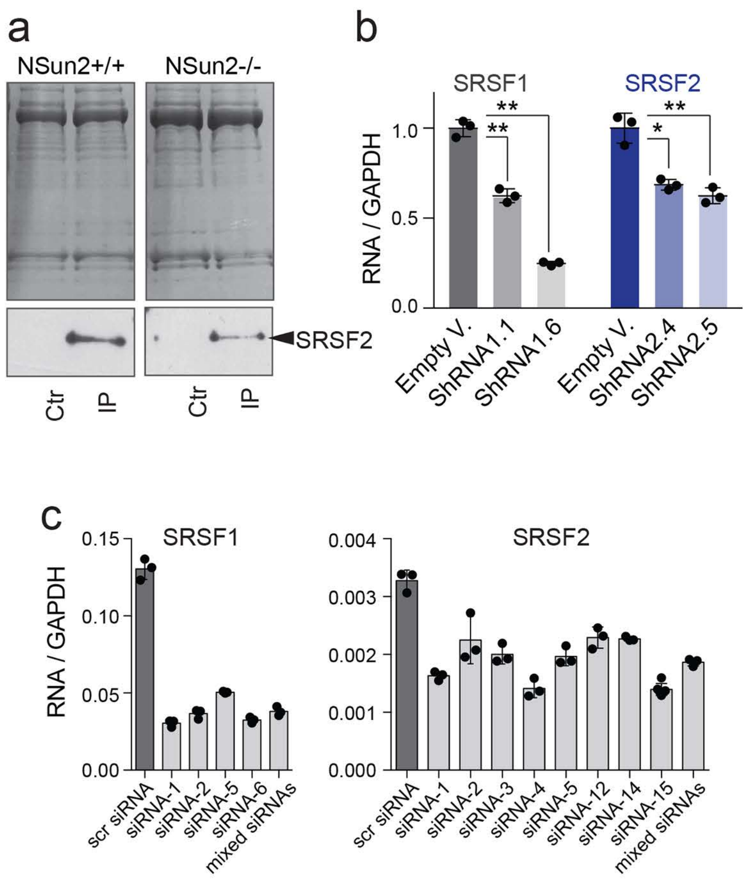


**Supplementary Figure 3. Biogenesis of svRNA4 is altered in the absence of SRSF2.** (**a**) Coomassie stained gel (upper panels) and immunoprecipitation (IP) detecting SFSR2 in *NSUN2*+/+ and *NSUN2*-/- lysates (lower panels). Ctr: Rabbit serum conjugated with Dynabeads. (**b**) SRSF1 and 2 RNA levels in *NSUN2*-/- human fibroblasts infected with shRNA empty vector (Empty V.), SRSF1 specific shRNAs (1.1 and 1.6) and SRSF2 specific shRNAs (2.4 and 2.5). Values were normalized to GAPDH. **p<0.01, *p<0.05 ANOVA. (**c**) Knock-down of SRSF1 (left hand panel) and SRSF2 (right hand panel) using different siRNA constructs. Shown are mean values of RNA levels versus GAPDH. Error bars indicate s.d. (n=3 qRT-PCRs). Source data are provided as a Source Data file.

# Supplementary Figure 4

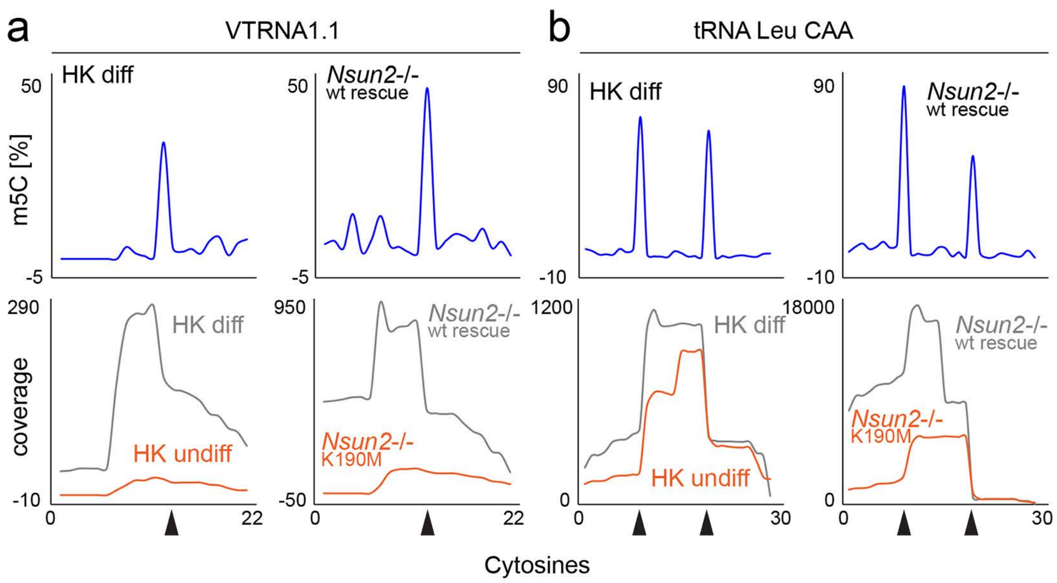


**Supplementary Figure 4. Coverage of NSUN2-dependent methylation.** (**a, b**) Methylation level (n=5 bisulfite conversion experiments) (upper panels) and coverage (reads) of the sites (lower panels) in VTRNA1.1 (a) and tRNA Leu CAA (b) in undifferentiated (undiff) and differentiated (diff) primary human keratinocytes (HK) (left hand panels) and *NSUN2*-/- human dermal fibroblasts rescued with the wild-type (wt) or enzymatic dead (K190M) construct of NSUN2.

# Supplementary Figure 5

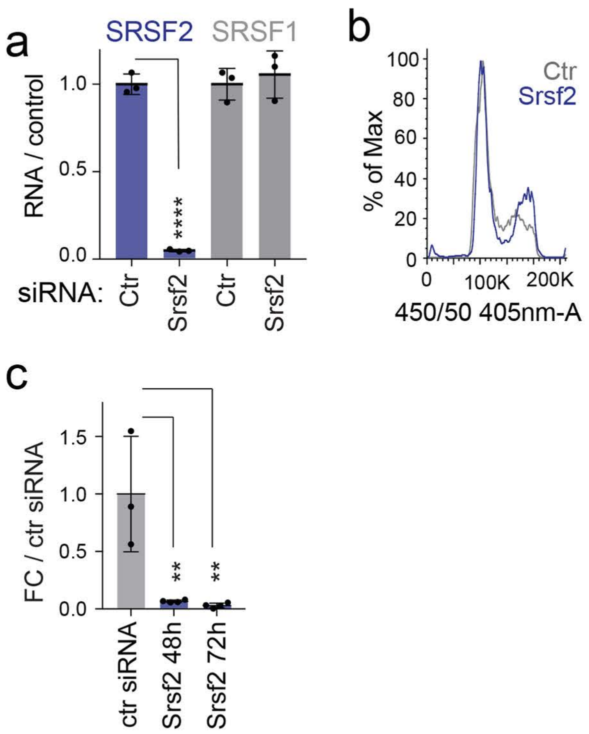


**Supplementary Figure 5. SRSF2 is required for cell divisions.** (**a**) RNA expression levels of *Srsf1* and *Srsf2* after transfection of a control siRNA (ctr) or a siRNA for *Srsf1* and *Srsf2*. Error bars represent s.d. (n =3 qRT-PCRs). ****p<0.0001 unpaired t-test. (**b**) Cell cycle profile of primary HK after 24 hours of *Srsf2* knock-down compared to control siRNA (Ctr) treated cells in high calcium medium. (**c**) RNA expression levels of *Srsf2* after transfection of a control siRNA (ctr) or a siRNA for *Srsf2*. Error bars represent s.d. (n = 3-4 qRT-PCRs). **p<0.01 One-way ANOVA. Source data are provided as a Source Data file.
