# Supplementary material for: Loss of 5-methylcytosine alters the biogenesis of vault-derived small RNAs to coordinate epidermal differentiation
Source: Nat Commun. 2019 Jun 11;10:2550. doi: 10.1038/s41467-019-10020-7 (PMC6560067; doi:10.1038/s41467-019-10020-7)
Supplement: Supplementary file 3 — Description of Additional Supplementary Files [file 41467_2019_10020_MOESM3_ESM.pdf]

Description of Additional Supplementary Files

File Name: Supplementary Data 1
Description: 1: Methylation levels after re-expression of NSUN2 (NSUN1-5), an enzymatic dead version of NSUN2 (K190M1-5), or the empty vector control (Empty1-5).

File Name: Supplementary Data 2
Description: Complete raw data of RNA pull-down and quantitative mass spectrometry from replicate 1.

File Name: Supplementary Data 3
Description: Complete raw data of RNA pull-down and quantitative mass spectrometry from replicate 2.

File Name: Supplementary Data 4
Description: Gorilla Gene Ontology analyses for 144 identified proteins (using all identified proteins from second replicate as background).

File Name: Supplementary Data 5
Description: Methylation levels in undifferentiated and differentiated keratinocytes. Shown are all sites with >100 coverage and >20% methylation in at least one condition.

File Name: Supplementary Data 6
Description: NSUN2-dependent methylation changes in undifferentiated and differentiated keratinocytes. Shown are all sites with >100 coverage and >20% m5C in at least one condition.
