# Supplementary material for: Loss of 5-methylcytosine alters the biogenesis of vault-derived small RNAs to coordinate epidermal differentiation
Source: Nat Commun. 2019 Jun 11;10:2550. doi: 10.1038/s41467-019-10020-7 (PMC6560067; doi:10.1038/s41467-019-10020-7)
Supplement: Supplementary file 10 — Reporting Summary [file 41467_2019_10020_MOESM10_ESM.pdf]

nature research

Corresponding author(s): Michaela Frye and Gracjan Michlewski

Last updated by author(s): Mar 5, 2019

Reporting Summary

Nature Research wishes to improve the reproducibility of the work that we publish. This form provides structure for consistency and transparency in reporting. For further information on Nature Research policies, see Authors & Referees and the Editorial Policy Checklist.

Statistics

For all statistical analyses, confirm that the following items are present in the figure legend, table legend, main text, or Methods section.

n/a	Confirmed	
☐	☒	The exact sample size (*n*) for each experimental group/condition, given as a discrete number and unit of measurement
☐	☒	A statement on whether measurements were taken from distinct samples or whether the same sample was measured repeatedly
☐	☒	The statistical test(s) used AND whether they are one- or two-sided<br>*Only common tests should be described solely by name; describe more complex techniques in the Methods section.*
☐	☒	A description of all covariates tested
☒	☐	A description of any assumptions or corrections, such as tests of normality and adjustment for multiple comparisons
☐	☒	A full description of the statistical parameters including central tendency (e.g. means) or other basic estimates (e.g. regression coefficient) AND variation (e.g. standard deviation) or associated estimates of uncertainty (e.g. confidence intervals)
☐	☒	For null hypothesis testing, the test statistic (e.g. *F*, *t*, *r*) with confidence intervals, effect sizes, degrees of freedom and *P* value noted<br>*Give P values as exact values whenever suitable.*
☒	☐	For Bayesian analysis, information on the choice of priors and Markov chain Monte Carlo settings
☒	☐	For hierarchical and complex designs, identification of the appropriate level for tests and full reporting of outcomes
☒	☐	Estimates of effect sizes (e.g. Cohen's *d*, Pearson's *r*), indicating how they were calculated

Our web collection on statistics for biologists contains articles on many of the points above.

Software and code

Policy information about availability of computer code

Data collection	No software was used.
Data analysis	Codes are deposited on GitHub. Bioinformatic analyses to calculate m5C levels included Trim Galore! (v0.4.0). Alignment to the reference genome was done using Bismark (v0.14.4). Display items and statistical analyses were done in Graphpad Prism and Excel. GO analyses were done using GOrilla (http://cbl-gorilla.cs.technion.ac.il/). Quantification of Western Blots was performed using ImageJ.

For manuscripts utilizing custom algorithms or software that are central to the research but not yet described in published literature, software must be made available to editors/reviewers. We strongly encourage code deposition in a community repository (e.g. GitHub). See the Nature Research guidelines for submitting code & software for further information.

Data

Policy information about availability of data

All manuscripts must include a data availability statement. This statement should provide the following information, where applicable:

- Accession codes, unique identifiers, or web links for publicly available datasets
- A list of figures that have associated raw data
- A description of any restrictions on data availability

Accession codes to all sequencing data are provided. Raw data are provided as supplementary datasets. Source data are provided for all panels.

Field-specific reporting

Please select the one below that is the best fit for your research. If you are not sure, read the appropriate sections before making your selection.

☒ Life sciences ☐ Behavioural & social sciences ☐ Ecological, evolutionary & environmental sciences

For a reference copy of the document with all sections, see nature.com/documents/nr-reporting-summary-flat.pdf

Life sciences study design

All studies must disclose on these points even when the disclosure is negative.

Sample size	The sample sizes for each experiment is stated in the figure legends and methods section and was 3 to 5. We calculated the means of the two independent groups based on the assumption that the data follow a normal, i.e. Gaussian distribution. Our null hypothesis is H0: µ sample 1 = µ sample 2; H1: µ sample 1 ≠ µ sample 2. Using G*power 3.1 and assuming we use a Student's unpaired t-test, a sample size of 3-5 will give us approximately 80% power (alpha = 0.05, two-tail) to detect an effect size of 2. For each experimental situation, we calculated mean and standard deviation. The significant differences between the sample populations depended on the comparison and was estimated using the unpaired student's t-test or ANOVA.
Data exclusions	No data points were excluded.
Replication	Data or findings that were not replicated were not included in this study.
Randomization	Not relevant for this study as no samples from organisms or participants were taken. All experiments included the corresponding controls (e.g. scrambled siRNA, random shRNA sequence, empty vector transfections, or point mutated RNA sequences).
Blinding	No blinding was possible as the samples were labeled when controls (see above) were used.

Reporting for specific materials, systems and methods

We require information from authors about some types of materials, experimental systems and methods used in many studies. Here, indicate whether each material, system or method listed is relevant to your study. If you are not sure if a list item applies to your research, read the appropriate section before selecting a response.

Materials & experimental systems

n/a	Involved in the study	
☐	☒	Antibodies
☐	☒	Eukaryotic cell lines
☒	☐	Palaeontology
☒	☐	Animals and other organisms
☒	☐	Human research participants
☒	☐	Clinical data

Methods

n/a	Involved in the study	
☒	☐	ChIP-seq
☐	☒	Flow cytometry
☒	☐	MRI-based neuroimaging

Antibodies

Antibodies used	NSUN2 (Met-A, rabbit), SRSF2 (ab11826 / mouse mAb/ Abcam), SRSF1 (32-4500 / mouse mAb / thermos fisher scientific), Tubulin (clone DM1A / mouse mAb / sigma), KRT10 (Covance - PRB-159P), OVOL1 (Ab65023 /Rabbit poly Ab / Abcam), hnRNPA1 (D21H11 / Rabbit mAb/ cell signaling), PUS7 ((4A2) ab118039 / mouse mAb / abcam)
Validation	NSUN2 antibody was validated in: Frye, M. & Watt, F. M. The RNA methyltransferase Misu (NSun2) mediates Myc-induced proliferation and is upregulated in tumors. Curr Biol 16, 971-981 (2006). SRSF1 and 2 antibodies were validated in this study by showing that the band disappeared in the siRNA- or shRNA-treated samples (Figure 3d and Figure 5b). KRT10 is validated by IF to stain the correct epidermal layers. hnRNPA1 was validated in Choudhury, N. R. & Michlewski, G. Quantitative identification of proteins that influence miRNA biogenesis by RNA pull-down-SILAC mass spectrometry (RP-SMS). Methods, doi:10.1016/j.ymeth.2018.06.006 (2018). OVOL1 and PUS7 by size in Western Blot.

Eukaryotic cell lines

Policy information about cell lines

Cell line source(s)	Human dermal fibroblasts are described in Martinez, F. J. et al. Whole exome sequencing identifies a splicing mutation in NSUN2 as a cause of a Dubowitz-like syndrome. J Med Genet 49, 380-385, doi:10.1136/jmedgenet-2011-100686 (2012).

	Human primary keratinocytes (ZHC-1116) were purchased from Cellworks (https://www.cellworks.co.uk). H9 were obtained by WiCell (https://www.wicell.org), HEK293 were obtained from ATCC (https://www.lgcstandards-atcc.org).
Authentication	Human dermal fibroblasts are validated by sequencing and Western blotting for NSUN2. Primary human keratinocytes are validated by marker expression (epidermis-specific keratins). H9 cells are validated by expression of pluripotency factors and morphology.
Mycoplasma contamination	All cell lines tested negatively for mycoplasma.
Commonly misidentified lines (See ICLAC register)	HEK293 and Hela, only used in assays that is unaffected by cell identity (eg SILAC).

Flow Cytometry

Plots

Confirm that:

- [ ] The axis labels state the marker and fluorochrome used (e.g. CD4-FITC).
- [ ] The axis scales are clearly visible. Include numbers along axes only for bottom left plot of group (a 'group' is an analysis of identical markers).
- [ ] All plots are contour plots with outliers or pseudocolor plots.
- [x] A numerical value for number of cells or percentage (with statistics) is provided.

Methodology

Sample preparation	Cells were washed in PBS and collected with Trypsin-EDTA (1:1 in PBS). Cells were fixed by resuspending in ice-cold 70% ethanol. Before processing cells were centrifuged at 12000g for 5 minutes and resuspended in 3mL PBS with DAPI 1:3000
Instrument	LSRFortessa Flow Cytometer (BD Bioseinces)
Software	FCS express6 (DeNovo Software)
Cell population abundance	All singlet events were analysed. (no post-sort fractioning was performed in this experiment)
Gating strategy	Fluorescence of each sample was measured at 450/50 405nm. All samples were gated uising forward versus side scatter to eliminate debris.

- [ ] Tick this box to confirm that a figure exemplifying the gating strategy is provided in the Supplementary Information.
